# Repeated Lineage Switches in an Elderly Case of Refractory B-Cell Acute Lymphoblastic Leukemia With *MLL* Gene Amplification: A Case Report and Literature Review

**DOI:** 10.3389/fonc.2022.799982

**Published:** 2022-03-23

**Authors:** Reina Takeda, Kazuaki Yokoyama, Tomofusa Fukuyama, Toyotaka Kawamata, Mika Ito, Nozomi Yusa, Rika Kasajima, Eigo Shimizu, Nobuhiro Ohno, Kaoru Uchimaru, Rui Yamaguchi, Seiya Imoto, Satoru Miyano, Arinobu Tojo

**Affiliations:** ^1^ Department of Hematology/Oncology, Research Hospital, The Institute of Medical Science, The University of Tokyo, Tokyo, Japan; ^2^ Division of Cellular Therapy, The Institute of Medical Science, The University of Tokyo, Tokyo, Japan; ^3^ Division of Molecular Therapy, The Institute of Medical Science, University of Tokyo, Tokyo, Japan; ^4^ Department of Applied Genomics, Research Hospital, Institute of Medical Science, University of Tokyo, Tokyo, Japan; ^5^ Division of Health Medical Data Science, Health Intelligence Center, Institute of Medical Science, University of Tokyo, Tokyo, Japan; ^6^ Molecular Pathology and Genetics Division, Kanagawa Cancer Center Research Institute, Yokohama, Japan; ^7^ Laboratory of DNA Information Analysis, Human Genome Center, Institute of Medical Science, University of Tokyo, Tokyo, Japan; ^8^ Department of Hematology, Kanto Rosai Hospital, Kanagawa, Japan; ^9^ Laboratory of Tumor Cell Biology, Department of Computational Biology and Medical Science, Graduate School of the Frontier Science, The University of Tokyo, Tokyo, Japan

**Keywords:** lineage switch, B-ALL, AML – acute myeloid leukaemia, MPAL – mixed phenotypic acute leukaemia, MLL, gene amplicaiton, TP53, monosomy 17

## Abstract

Lineage switches in acute leukemia occur rarely, and the underlying mechanisms are poorly understood. Herein, we report the case of an elderly patient with leukemia in which the leukemia started as B-cell acute lymphoblastic leukemia (B-ALL) and later changed to B- and T-cell mixed phenotype acute leukemia (MPAL) and acute myeloid leukemia (AML) during consecutive induction chemotherapy treatments. A 65-year-old woman was initially diagnosed with Philadelphia chromosome-negative B-ALL primarily expressing TdT/CD34/HLA-DR; more than 20% of the blasts were positive for CD19/CD20/cytoplasmic CD79a/cytoplasmic CD22/CD13/CD71.The blasts were negative for T-lineage markers and myeloperoxidase (MPO). Induction chemotherapy with the standard regimen for B-ALL resulted in primary induction failure. After the second induction chemotherapy regimen, the blasts were found to be B/T bi-phenotypic with additional expression of cytoplasmic CD3. A single course of clofarabine (the fourth induction chemotherapy regimen) dramatically reduced lymphoid marker levels. However, the myeloid markers (e.g., MPO) eventually showed positivity and the leukemia completely changed its lineage to AML. Despite subsequent intensive chemotherapy regimens designed for AML, the patient’s leukemia was uncontrollable and a new monoblastic population emerged. The patient died approximately 8 months after the initial diagnosis without experiencing stable remission. Several cytogenetic and genetic features were commonly identified in the initial diagnostic B-ALL and in the following AML, suggesting that this case should be classified as lineage switching leukemia rather than multiple simultaneous cancers (i.e., *de novo* B-ALL and *de novo* AML, or primary B-ALL and therapy-related myeloid neoplasm). A complex karyotype was persistently observed with a hemi-allelic loss of chromosome 17 (the location of the *TP53* tumor suppressor gene). As the leukemia progressed, the karyotype became more complex, with the additional abnormalities. Sequential target sequencing revealed an increased variant allele frequency of *TP53* mutation. Fluorescent *in situ* hybridization (FISH) revealed an increased number of *mixed-lineage leukemia* (*MLL*) genes, both before and after lineage conversion. In contrast, FISH revealed negativity for *MLL* rearrangements, which are well-known abnormalities associated with lineage switching leukemia and MPAL. To our best knowledge, this is the first reported case of acute leukemia presenting with lineage ambiguity and *MLL* gene amplification.

## Introduction

A lineage switch from acute lymphoblastic leukemia (ALL) to acute myeloid leukemia (AML) and *vice versa* is a rare event observed during the relapse of acute leukemia and is associated with dismal clinical outcomes (1–3). Lineage conversion is found more frequently in pediatric (6-9% prevalence) than in adult patients (1, 2). A high prevalence of lineage switching has been reported in infants younger than 1 year of age presenting with acute leukemia, including neonates aged younger than 1 month with congenital acute leukemia (2, 3). However, a few cases of adult lineage switching leukemia have been reported, and even fewer cases have been reported in the elderly (2, 4–6). The majority of cases of lineage switching leukemia harbor *mixed-lineage leukemia* (*MLL*, also known as *MLL1* and *KMT2A*) gene rearrangements (2–5). Several hypotheses have been proposed to explain the lineage switches and ambiguity occurring in leukemia. For instance, genetic and/or epigenetic dysregulation of lineage-specific transcription factors (e.g., *PU.1*, *CEBPA*, *PAX5*) may rewrite the differentiation programs of bi-, tri-, and oligo-potential leukemic clones (2, 7, 8). Therapies may facilitate the selection of subclones that are better equipped for survival (7, 9, 10). The tumor microenvironment could also influence the choice of cell lineage for leukemia cells *via* cytokines and metabolic parameters (2, 7). The hematopoietic microenvironment and stroma changes during developmental stages; thus, the fetal liver and adult bone marrow may differentially contribute to defining leukemia lineage fates (11). A leukemia initiating clone with a primitive cell of origin may be multipotent, producing a variety of hematopoietic lineages (2, 7). However, the precise mechanisms underlying leukemia lineage switches remain unclear.

Gene amplification occurs in various types of malignancies. Amplified genes often lead to the overexpression of proto-oncogenes, resulting in aggressive tumor development and poor prognoses (12, 13). Compared to solid tumors, gene amplification is rarely observed in hematological malignancies. The frequency of cytogenetically detectable gene amplification is approximately 1% in AML (12–14). While *MYC* is the most common amplicon gene, *MLL* gene amplification is also found in myeloid malignancies, including myelodysplastic syndrome (MDS) and AML (12–15).

Herein, we present the case of an elderly patient with Philadelphia chromosome (Ph)-negative B-cell ALL (B-ALL). The leukemia changed immunophenotypes and lineages in rapid succession, which is unusual in elderly patients. The leukemia was negative for any *MLL* rearrangement, which often occurs in leukemia cases with lineage ambiguity. Instead, *MLL* gene amplification was observed. The multi-drug resistance as well as treatment failure without achieving durable remission seen in this case could be related to the patient’s highly complex karyotype and the presence of *TP53* alterations. Herein, we report a newly identified case of *MLL*-amplified leukemia with high lineage ambiguity, along with next-generation sequencing (NGS) results and a comprehensive literature review.

## Case Presentation

A 65-year-old woman was referred to our hospital due to pancytopenia and disseminated intravascular coagulation (DIC). She complained of a 2-month history of fatigue. Her performance status (Eastern Cooperative Oncology Group) score upon examination was 0. She had a history of well-controlled hypertension and mild liver dysfunction, which was treated with ursodeoxycholic acid. There was no cancer or blood disease in her family history.

**Figure 1:**
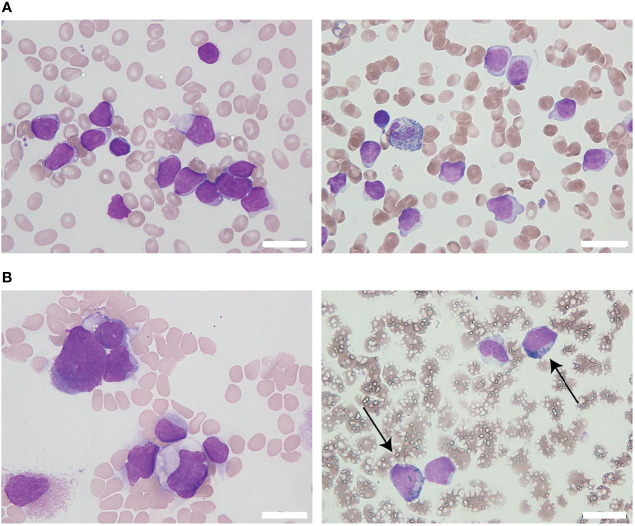
The appearance of leukemia cells in the present case. **(A)** At initial diagnosis, the bone marrow (BM) was filled with morphologically monotonous myeloperoxidase (MPO)-negative leukemic blast cells (May–Giemsa stain [left]; MPO stain [right]; original magnification, 1,000×). **(B)** After the fourth regimen of chemotherapy, the BM presented with an increased number of MPO-positive leukemia blasts (arrows; May–Giemsa stain [left]; MPO stain [right]; original magnification, 1,000×). White bars in the right bottom portion of the figure represent 20 µm units.

On admission, peripheral blood examination revealed a white blood cell (WBC) count of 2.0 × 10^9^/L, with 4.5% neutrophils and 11.0% myeloperoxidase (MPO)-negative blasts. Anemia (hemoglobin level, 6.7 g/dL) and thrombocytopenia (platelet count, 38 × 10^9^/L) were also detected. Her lactic dehydrogenase (LDH) level was slightly elevated at 252 IU/L (reference range, 105-211 IU/L). A mild elevation of biliary enzymes and DIC were observed as well. Physical examination and computed tomography (CT) evaluations detected no evidence of hepatosplenomegaly or lymphadenopathy. Bone marrow (BM) aspiration showed infiltration of MPO-negative blasts in 94% of the nucleated cell count (NCC) (**Figure 1A**). Flow cytometric analysis revealed that most of the blast cells were CD45 dim positive as well as highly positive for CD34, TdT, and HLA-DR. More than 20% of the blast cells were positive for CD19, CD20, cytoplasmic CD79a (cyCD79a), cytoplasmic CD22, CD13, and CD71. A small fraction of the population (up to approximately 10%) was positive for CD2, CD10, CD33, and CD56, whereas test results for CD3, CD4, CD5, CD7, CD8, CD14, CD16, CD41, MPO, and glycophorin A were negative. Cytogenetic analysis identified a complex karyotype with monosomy X, monosomy 5, and monosomy 17 (**Table 1**). A screening analysis for leukemia chimeric genes was negative for *BCR-ABL* (major, minor, or micro), *E2A-PBX*, *TEL-AML1*, *MLL-AF4*, and *MLL-AF9*. These results confirmed a diagnosis of Ph-negative B-ALL. The previously validated unfavorable prognostic factors for adult ALL seen in this case were older age and a complex karyotype (16–18).

**Table 1:** Changes in karyotype and *TP53* mutation frequency of the present case.

Days after diagnosis, and aim of analysis		Leukemia phenotype	Karyotype [number of cells] and the frequency of cytogenetically abnormal cells (%)		*TP53* mutation VAF (%)	*MLL* amplification (%)
0	Diagnosis (BM)	B-ALL	44, X, -X, add (1) (p13), add (2) (q21), -4, -5, -10, del(11)(q)?, -12, -14, -17, -18, +r1, +mar1, +mar2, +mar3, +mar4, +mar5 [3]/46, XX [4].	80 [16]/[20]	49.5	91.5
38	1st chemo evaluation (BM)	B-ALL	43, X, -X, add(1)(p13), add(2)(q21), -4, -5, -10, add(11)(q13), -12, -14, -17, -18, +mar1, +mar2, +mar3, +mar4, +mar5 [4]/44, idem, +r1 [3]/46, XX [11].	45 [9]/[20]	Not done	Not done
79	2nd chemo evaluation (BM)	B/T MPAL	43, X, -X, add(1)(p13), add(2)(q21), -4, -5, -10, add(11)(q13), -12, -14, -17, -18, +mar1, +mar2, +mar3, +mar4, +mar5 [2]/46, XX [5].	75 [15]/[20]	Note done	Not done
112	3rd chemo evaluation (BM)	B/T MPAL	44, X, -X, add(1)(p13), add(2)(q21), -4, -5, add(7)(q11.2), -10, add(11)(q13), -12, -14, -17, -18, +mar1, +mar2, +mar3, +mar4, +mar5, +mar6 [1]/87, idem x2, +5, +7, +7,-add(7) x2, -13, -mar3, -mar4, -mar5, -mar6 x2, +mar4 [1]/46, XX [6]	70 [14]/[20]	76.2	86.6
153	4th chemo evaluation (BM)	AML	45, X, -X, add(1)(p13), add(2)(q21), -4, -5, -8, -10, add(11)(q13), -12, -13, add(17)(p11.2), +mar1, +mar2, +mar3, +mar4, +mar5, +mar6 [1]/43, idem, -7, -add(17), +add(17)(p11.2), -mar6 [1]	100 [20]/[20]	94.1	64.7
198	5th chemo evaluation (PB)	AMML	43, X, -X, add(1)(p13), add(2)(q21), -4, -5, -8, -10, add(11)(q13), -12, -13, -17, +mar5 [1]/46, XX [2]	88.2 [15]/[17]	Not done	43.4
219	6thc chemo evaluation (PB)	AMML	43, X, -X, add(1)(p13), add(2)(q21), -4, -5, -7, -8, -10, add(11)(q13), -12, -13, -17, +6mar [1]	100 [10]/[10]	Not done	68.1
235	Died					

**Figure 2:**
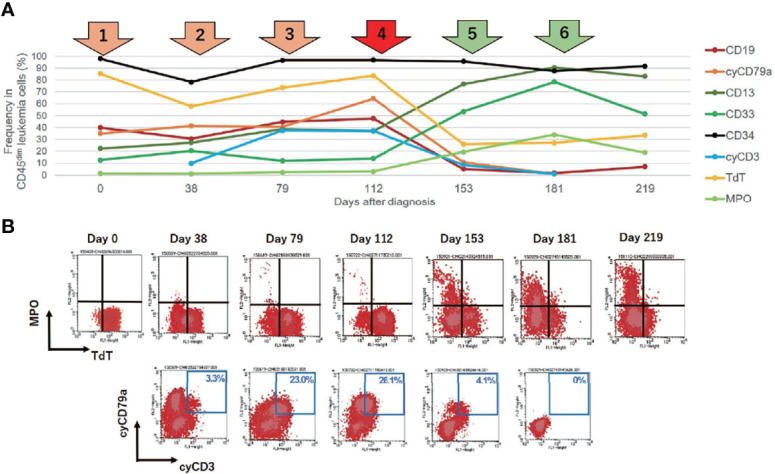
Changes in leukemia cell immunophenotypes in the present case. Flow cytometric analysis revealed that leukemia blast cells in this case evolved immunophenotypically during sequential intensive chemotherapy. The analyzed leukemia cells were taken from bone marrow samples, with the exception of days 181 and 219 (when samples were taken from peripheral blood). **(A)** Changes in the frequency of immunophenotypic markers in CD45 dim leukemia cells. The arrows above the graph represent each chemotherapy regimen. Orange and red arrows represent intensive chemotherapy regimens for B-cell acute lymphoblastic leukemia (B-ALL), whereas green arrows indicate treatment regimens for acute myeloid leukemia (AML). After clofarabine monotherapy, administered as a fourth chemotherapy regimen (red arrow), a dynamic change in lineage marker expression was observed. **(B)** Changes in the immunophenotypic plot of CD45 dim leukemia cells (upper row, myeloid [myeloperoxidase, MPO] *vs.* lymphoid [TdT]; bottom row, B-lymphoid [cyCD79a] *vs.* T-lymphoid [cyCD3]).

The patient initially received standard chemotherapy for adult Ph-negative B-ALL, comprised of daunorubicin, cyclophosphamide, vincristine, L-asparaginase, and prednisolone. Despite induction therapy, the BM was still occupied by leukemia cells, accounting for 79% of the NCC. This finding was considered to reflect primary induction failure. Remission was not achieved following two additional lines of conventional intensive chemotherapies for B-ALL, comprising cytarabine/etoposide/dexamethasone as well as methotrexate/cytarabine. Throughout these sequential chemotherapies, the leukemia cells were found to additionally express cytoplasmic CD3 (cyCD3), and the B/T bi-phenotypic leukemia population gradually expanded (**Figures 2A, B**). The karyotypes became more complex with extra abnormalities, including additional material of unknown origin (add) in chromosome 11q (**Table 1**). Since mixed phenotype leukemia (MPAL) often harbors *MLL*-fusion genes derived from 11q chromosomal translocations, we performed a fluorescent *in situ* hybridization (FISH) analysis evaluating *MLL* rearrangements. Interphase FISH analysis of the split signal of the *MLL* gene on chromosome 11q23 revealed that the evaluated leukemia cells were negative for *MLL* gene rearrangements, however, the majority of these cells possessed more than eight copies of the full-length *MLL* gene per nucleus. Based on data from immunophenotypic, karyotypic, and *MLL* FISH analyses, the patient’s leukemia was re-classified into MPAL, not otherwise specified (NOS) in accordance with the World Health Organization (WHO) 2016 classification guidelines (19, 20).

**Figure 3:**
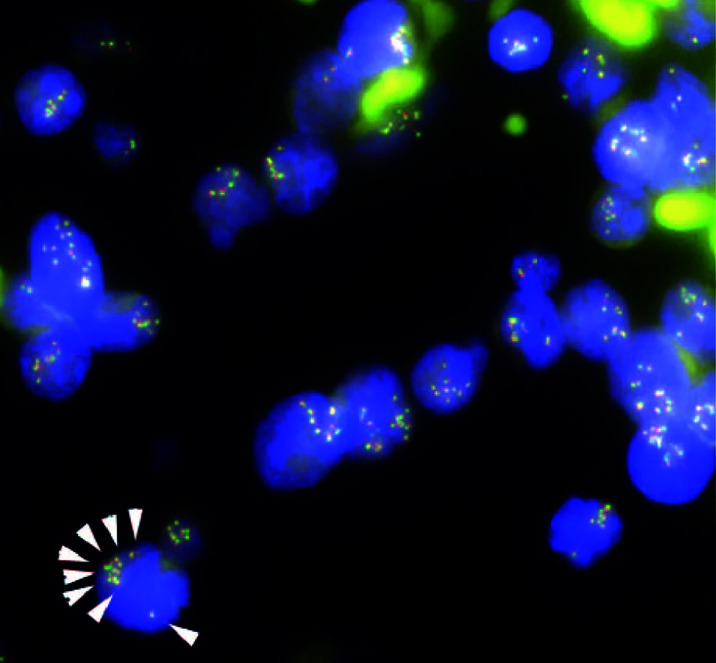
Fluorescence *in situ* hybridization (FISH) analysis for gene rearrangements involving in the *mixed lineage leukemia* (*MLL*) gene (11q23) in the present case. *MLL* FISH data at the time of the initial B-cell acute lymphoblastic leukemia (B-ALL) diagnosis. The full-length *MLL* gene is shown *via* a yellow signal, whereas the rearranged *MLL* gene is shown *via* a pair of split signals colored green and red. A representative cell in the lower left portion of the figure shows eight full-length *MLL* signals (white arrows) in the nucleus. At the time of initial diagnosis, 91.5% of the interphase cells had at least eight copies of non-rearranged *MLL* genes. No *MLL* split signals generated by gene rearrangements were observed throughout the disease course.

Given the unexpected result of an increased number of *MLL* gene copies, we retrospectively investigated the status of the *MLL* gene at the time of the initial diagnosis *via* the FISH evaluation. Due to the lack of fresh cell samples, we evaluated paraffin-embedded BM specimens that were collected on admission and found *MLL* amplification without *MLL* translocation in more than 90% of the interphase cells in the initial diagnostic B-ALL cells (**Figure 3**). The *MLL* FISH signals were separately distributed and were not clustered in one location, suggesting that *MLL* loci were spread across multiple regions and chromosomes. Since the karyotyping analyses showed no chromosomal alterations directly affecting 11q23, and neither well-known hallmarks of gene amplification such as homogenously staining region (hsr) and double minutes (dmin), the aberrantly amplified *MLL* genes in this case were presumed to have been caused by multiple marker chromosomes (mar) as well as by a ring chromosome (r) (12, 13).

To overcome the refractoriness and resistance to multiple drugs of this disease presentation, clofarabine, a second-generation purine analog, was administered as a single agent in a fourth induction chemotherapy regimen. Nevertheless, leukemia cells reappeared in the peripheral blood and proliferated rapidly. A BM examination revealed hypocellular BM with infiltration of blast cells in 66% of the NCC. Unlike in the previous evaluation, more than 3% of the blast cells were positive for MPO staining (**Figure 1B**). Flow cytometric analysis showed that most of the leukemia cells were positive for CD13 and CD33, but were negative for CD19, cyCD79a, and cyCD3. The rapid increase in MPO expression and the dramatic loss of TdT expression indicated a dynamic immunophenotypic change from a lymphoid to a myeloid lineage (**Figures 2A, B**). Karyotype analysis revealed that all examined cells were abnormal and had extremely complex karyotypes. Most cytogenetic abnormalities had persisted since the time of diagnosis (**Table 1**), and new chromosomal aberrations (e.g., monosomy 8 and monosomy 13) were simultaneously detected as well. *MLL* FISH analyses demonstrated the persistence of *MLL* gene amplification without rearrangements. These findings led to the conclusive diagnosis of acute myeloid leukemia (AML), as a result of leukemia progression with a lineage conversion from MPAL, NOS (B/T bi-phenotypic leukemia).

After conversion to the myeloid lineage, the patient was treated with two lines of therapies commonly used for AML. The first line of therapy was a combination chemotherapy regimen consisting of cytarabine, anthracycline, and granulocyte-colony stimulating factor (G-CSF), and the second line of therapy was a monotherapy with gemtuzumab ozogamicin, a calicheamicin-conjugated humanized anti-CD33 monoclonal antibody. However, the leukemia did not respond to either treatment, resulting in disease deterioration with a rising cell subpopulation. Morphological and flow cytometric analyses identified this new subpopulation to be monoblastic. The cells in this monoblastic population showed high expression levels of CD13, CD33, CD14, CD45, and HLA-DR, and more than 20% of the monoblastic cells were positive for CD4, CD16, CD34 and MPO. Almost eight months (235 days) after the initial diagnosis of B-ALL and approximately three months after the lineage switch to AML, the patient died of multiple intracranial hemorrhages due to severe DIC associated with the drastic progression of AML.

To gain insight into the molecular mechanisms responsible for treatment resistance and lineage switching in the current case, we performed a targeted deep sequencing analysis. We analyzed the patient’s BM samples, which were collected at three different time points during the disease course. One BM sample was collected at the time of the initial diagnosis (day 0) and the other two samples were taken before and after a dynamic lineage change (at days 112 and 153, respectively). NGS was performed using extracted DNA from each BM sample *via* the TruSight Myeloid Panel on the MiSeq platform (Illumina, San Diego, CA, USA). This panel allowed for the effective detection of myeloid neoplasm-associated hotspot mutations in the following 54 genes: *ABL1*, *ASXL1*, *ATRX*, *BCOR*, *BCORL1*, *BRAF*, *CALR*, *CBL*, *CBLB*, *CBLC*, *CDKN2A*, *CEBPA*, *CSF3R*, *CUX1*, *DNMT3A*, *ETV6*/*TEL*, *EZH2*, *FBXW7*, *FLT3*, *GATA1*, *GATA2*, *GNAS*, *HRAS*, *IDH1*, *IDH2*, *IKZF1*, *JAK2*, *JAK3*, *KDM6A*, *KIT*, *KRAS*, *MLL*, *MPL*, *MYD88*, *NOTCH1*, *NPM1*, *NRAS*, *PDGFRA*, *PHF6*, *PTEN*, *PTPN11*, *RAD21*, *RUNX1*, *SETBP1*, *SF3B1*, *SMC1A*, *SMC3*, *SRSF2*, *STAG2*, *TET2*, *TP53*, *U2AF1*, *WT1*, and *ZRSR2*. Oral epithelial cells collected *via* buccal swabs served as germline controls. Bioinformatic analysis was performed using standard procedures (21, 22). The analysis demonstrated a somatic mutation in exon5 of the *TP53* gene (c.455dupC:p.P153Afs*28) in all three samples (which, as noted above, were collected at different time points). Interestingly, the allele frequency of this *TP53* frameshift mutation gradually increased from 49.5% at time of the initial diagnosis, to 76.2% at the end of the third chemotherapy regimen, and finally to 94.1% at the end of the fourth chemotherapy regimen (when lineage conversion into AML was observed) (**Table 1**). No additional somatic or germline mutations were detected. This study was approved by the Institutional Review Board of the Institute of Medical Science at the University of Tokyo. Written informed consent was obtained from the patient in accordance with the Declaration of Helsinki.

## Discussion

Herein, we describe the case of an elderly patient with acute lineage switching leukemia that transformed from B-ALL to AML. To comprehensively discuss the pathophysiology of this case, three unique features should be highlighted. The first is the high lineage ambiguity observed in this case presentation. The expression patterns of immunophenotypic markers in leukemia cells changed continuously throughout the disease course. The second feature of note is the high degree of genome instability observed in this case. More specifically, the karyotypes became more complex as the disease progressed. *MLL* gene amplification and *TP53* alterations (hemi-allelic loss and a *TP53* frameshift mutation) were present from the time of the initial diagnosis. The third characteristic of note is the severe refractoriness of the disease presentation. Multiple anti-tumor drugs failed to achieve durable remission within any of the administered chemotherapy regimens.

As shown in this case report, the immunophenotype and cell lineage changed repeatedly throughout the duration of the disease course. Although it would have been preferable to verify whether and how the status of the TCR and immunoglobulin reconstitutions changed during disease progression in order to comprehensively discuss and describe clonal evolution and the cell of origin, we are unfortunately unable to do so due to sample limitations. However, based on the persistent cytogenetic and molecular abnormalities detected in the G-band, FISH, and NGS analyses, we concluded that there were lineage conversions between clones derived from a common ancestor. The acute leukemia initially presented with B-lineage cells, with aberrant expression of an erythroid marker (CD71) and a myeloid marker (CD13). Subsequently, the leukemia evolved to become B/T bi-phenotypic. Next, a dynamic lineage switch occurred, and the leukemia transformed to a myeloid lineage. Finally, a new monoblastic population was identified. With the exception of the persistent and strong expression of CD34 (a cell surface marker for early-stage hematopoiesis), we found that the expression patterns of the immunophenotypic markers of leukemic cells were constantly changing. A series of flow cytometry data indicated that the leukemia-initiating cells in this case had the potential to express not only B-lymphoid markers (CD19, CD20, cyCD22, and CD79a), but also T-lymphoid (cyCD3), myeloid (MPO, CD13, and CD33), monocytic (CD14), and erythroid (CD71) markers. Therefore, in this case, the cell of origin may have transformed at the early stages of hematopoiesis, such as hematopoietic stem cells (HSCs) or multipotent progenitors (MPPs).

The precise mechanisms of lineage interconversion remain to be fully elucidated with respect to *MLL*-rearranged leukemia as well as other genotyped leukemias. A previous study showed that *MLL* rearrangements were prevalent in the almost 80% of pediatric B-ALL patients who experienced lineage conversion to AML after undergoing chemotherapies with or without hematopoietic stem cell transplantation (3). In addition, an increasing number of AML phenotypic relapses in patients with *MLL*-rearranged B-ALL have been reported, especially after undergoing CD19-targeting immunotherapies (including blinatumomab and chimeric antigen receptor [CAR] T-cell therapy) (9, 10, 23, 24). These clinical observations support the hypothesis that *MLL*-fusion leukemia is prone to lineage ambiguity. Considering that the overexpression of the wild-type MLL protein produced by *MLL* gene amplification shares some target genes (e.g., *HOXA*, *MEIS1*) as well as an epigenetic regulatory system (e.g., histone H3K4 trimethylation) with MLL fusion proteins (13, 25–28), *MLL* amplification may potentially play a role in leukemia lineage plasticity. Nevertheless, the differences and redundancies between *MLL* rearrangements and *MLL* amplification remain controversial. It is also unknown whether the *MLL* amplicon functions as a driver or a passenger in the process of leukemogenicity. Additional studies are warranted to investigate the biological significance of *MLL* amplification in leukemia.

*MLL* gene amplification is found in approximately 1% of AML cases (12–14, 29). Unlike the broad immunophenotypic repertoire of *MLL*-rearranged leukemias, *MLL*-amplified leukemia has been reported mostly in myeloid cases, including AML, MDS, and therapy-related myeloid neoplasms (t-MNs) previously treated with alkylating agents (12–15, 25, 26, 28–30). B-ALL with *MLL* amplification is extremely rare, with only a few cases described in the literature to date (29–31). Moreover, to our best knowledge, no cases of mixed lineage leukemia (MPAL) or of lineage switching leukemia with *MLL* amplification have been reported. Older age, DIC, therapy resistance, and poor outcomes were common characteristics of the previously reported cases of AML presenting with *MLL* amplification (12, 13, 15). These clinical features matched well with the present case. Interestingly, some genomic features that appeared in our case also seemed to be associated with *MLL* amplification. For example, previous cases of *MLL*-amplified AML/MDS almost always displayed highly complex karyotypes and frequently contained -5/del(5q), del(-7q), -17/del(17p), and -18 abnormalities (12, 13, 15, 25). In most *MLL*-amplified cases reported to date, karyotypes were reported to become more complex within short intervals (13, 15). These cytogenetic features were also observed in this case.

*Tumor protein p53* (*TP53*) is a tumor suppressor gene located on the short arm of chromosome 17. The p53 protein is an important transcription factor that regulates cell cycle arrest, apoptosis, and the DNA damage response. Due to its function as a guardian of chromosomal stability and genome integrity, the loss and/or mutational inactivation of p53 leads to genomic instability, thus resulting in oncogene amplification and aneuploidy (15). Cancers with p53 dysfunction are typically refractory to therapy and have unfavorable prognoses. Compared with *TP53* mutations in solid tumores (which are detected at a rate of more than 50%), the loss of or mutations in the *TP53* gene occur relatively rarely in hematological malignancies (32, 33). Among newly diagnosed patients, monosomy 17 is estimated to occur in approximately 7% of adult Ph-negative B-ALL cases (34), as well as in approximately 6% of adult AML cases (35). Moreover, the incidences of *TP53* mutations at initial diagnosis is approximately 8% in adult Ph-negative B-ALL patients (33, 36), and is approximately 8% in adult AML patients (32), respectively. In contrast, when the entity is limited to *MLL*-amplified AML/MDS, the frequency of monosomy 17 becomes remarkably higher and is estimated to reach 38% (13). Moreover, regardless of the presence or absence of cytogenetic abnormalities involving chromosome 17, the *TP53* gene is often mutated in *MLL*-amplified AML/MDS (13, 15, 25, 29). One previous study revealed that 94% of AML/MDS cases with 11q/*MLL* amplification carried *TP53* mutations (25). Hence, the loss of functional p53 may be critical to the etiology and progression of this *MLL*-amplified leukemia, including our currently reported case (25, 29).

The *TP53* gene mutation in the present case was a frameshift mutation in the DNA-binding domain that occurred concurrently with monosomy 17 since the time of the initial diagnosis. The vast majority of *TP53* mutations in malignancies (including AML and ALL) are found in the DNA binding domain (encoded by exons 5–8), with hot spots located at six particular amino acid residues, (R175, G245, R248, R249, R273, and R282) (32, 33). These are predominantly missense alternations resulting in mutant p53 proteins with one substituted amino acid; this abrogates DNA binding ability and impairs transcriptional activity (33, 37). As knowledge of *TP53* mutations in leukemogenesis has expanded in recent years, the mutant p53 protein has been found to not only plays a role as a typical tumor suppressor gene through loss-of-function alternations, but also potentially acts in a dominant negative or a gain-of-function manner (depending on mutated residues, the status of the remaining wild-type allele, and the context of the presenting cell types) (32, 37–39). *TP53* alterations are usually monoallelic. During disease progression, the remaining *TP53* wild-type gene in the second allele is also altered or lost due to mutation, monosomy of chromosome 17p/17, or loss of heterozygosity (LOH) (33) In contrast to these missense mutations, it is still unclear whether *TP53* frameshift mutations, as in our case, can be translated into C-terminus truncated mutant proteins. In addition, even though truncated p53 proteins exist, their biological functions are also still unclear. Additional investigation is warranted to clarify these points.

Lineage switching in leukemia generally occurs either at the first or second relapse (2). In contrast, lineage conversion during induction therapy without or prior to durable remission is atypical and is limited to a few reports of childhood cases (2, 3, 40–42). The clinical outcomes of leukemia cases where lineage switching occurs earlier than remission appear to be devastating. All reported patients, including our adult case, died within several months of the initial diagnosis (2, 41, 42). Currently, there is no standard recommended therapy for lineage switching leukemia and MPAL. Therefore, physicians often face challenges in the proper choice of therapy for ambiguous lineage leukemia as well as with regard to performing differential diagnoses. According to a recent retrospective study, allogenic hematopoietic stem cell transplantation (alloHSCT) in the first remission improves outcomes in adult MPAL (43). In addition, studies report that certain targeted therapies may be beneficial for child or young adult MPAL patients (20). However, caution should be exercised when using targeted therapies, as there is a theoretical risk of propagating non-targeted clones (43). For lineage switching leukemia, which patients with an extremely devastating prognosis, there have only been a few reports to date of cases experiencing long-term remission. For example, a case report of an infant patient with lineage switching leukemia showed that administering alloHSCT as a consolidation therapy following remission resulted in well-controlled disease without relapse over the course of almost 2 years of follow-up (24). In the present case, however, alloHSCT with reduced-intensity conditioning was not a viable option because of the patient’s advanced age and lack of remission.

To better understand the underlying pathophysiological mechanisms and develop an effective treatment strategy based on these mechanisms, animal disease models that recapitulate human leukemic characteristics are urgently needed. Recent studies using *MLL*-rearranged leukemia mouse models have shown that the hematopoietic niche, which changes during development and aging, plays an important role in determining the lineage of leukemic cells (11, 44, 45). Contrarily, several experimental studies using specific gene-expressing mouse models have revealed that the lineage output of leukemia can be more ambiguous depending on the hematopoietic hierarchy and the developmental and aging stage of the leukemic cells (44, 46–48). Another study using MPAL patient-derived xenograft (PDX) models also supported the relationship between lineage fate and the cell of origin by demonstrating that the cell of origin, which is rooted in immature hematopoietic progenitors, primes leukemia cells for lineage promiscuity in MPAL (20). Comprehensive analyses of the genome, epigenome, and transcriptome at the single-cell level would be helpful in elucidating the precise molecular mechanisms underlying clonal evolution and cell lineage plasticity. Given the rarity and heterogeneity of lineage conversion and MPAL, the utilization of NGS should be considered in each individual patient. This may enable not only effective disease profiling but also more comprehensive treatment recommendations for precision medicine.

In summary, we report that our case of refractory B-ALL with lineage conversion to AML, which occurred in an elderly patient, exhibited lineage ambiguity and genome instability. The cell of origin may have had multi-lineage potential. Treatment may exert selective pressure that drives clonal selectivity. Leukemia subclones harboring *TP53* mono-allelic loss, *TP53* frameshift mutation, and *MLL* amplification appeared to provide advantages in terms of proliferation and treatment resistance. For rare leukemia subtypes with no established therapies, effective pathophysiology-based therapy is urgently needed.

## Conclusion

Herein we presented a case of an elderly patient with Ph-negative B-ALL. Her leukemia was refractory to treatment and progressed with repetitive immunophenotypic changes. Dynamic lineage conversion from ALL to AML occurred after clofarabine monotherapy was provided as a fourth induction regimen. The karyotype was highly complex and rapidly increased in complexity. A monoallelic deletion and frameshift mutation in *TP53* gene accompanied by *MLL* gene amplification may have contributed to lineage plasticity and therapeutic resistance in this case. Additional accumulation of case studies, comprehensive clinical research, and basic and translational investigations are required to better understand this rare presentation of a form of leukemia that is currently without standard therapies or definitive medical guidelines. Our findings thereby guide and inform further research directions, medical guidelines, and effective clinical decision-making.

## Data Availability Statement

The datasets presented in this study can be found in online repositories. The names of the repository/repositories and accession number(s) can be found below: NBDC Human Database, JGAD000600, https://humandbs.biosciencedbc.jp/hum0318-v1.

## Ethics Statement

The studies involving human participants were reviewed and approved by the Institutional Review Board of the Institute of Medical Science, the University of Tokyo. The patients/participants provided their written informed consent to participate in this study.

## Author Contributions

RT, KY, TF, and AT designed the study. RT, KY, TF, TK, NO, and KU participated in patient treatment and analyzed clinical data. KY, MI, NY, RK, ES, RY, SI, SM, and AT performed next-generation sequencing and analyzed the data. RT drafted the manuscript, KY and TF helped draft the manuscript. AT supervised the work and helped draft the manuscript. All authors have read and approved of the final version of the manuscript.

## Conflict of Interest

The authors declare that the research was conducted in the absence of any commercial or financial relationships that could be construed as a potential conflict of interest.

## Publisher’s Note

All claims expressed in this article are solely those of the authors and do not necessarily represent those of their affiliated organizations, or those of the publisher, the editors and the reviewers. Any product that may be evaluated in this article, or claim that may be made by its manufacturer, is not guaranteed or endorsed by the publisher.
